# Single-Cell Sequencing Yields Insights in the Evolution of Foot-and-Mouth Disease Virus Persistent Infection

**DOI:** 10.3389/fcimb.2022.940906

**Published:** 2022-07-08

**Authors:** Yuncong Yuan, Xingran Wang, Jiadai Li, Lingling Han, Hang Du, Yidan Sun, Pu Yang, Zhou Zhou, Meijia Gu, Yang Lu, Chao Shen

**Affiliations:** ^1^ College of Life Sciences, Wuhan University, Wuhan, China,; ^2^ China Center for Type Culture Collection, Wuhan University, Wuhan, China; ^3^ Key Laboratory of Combinatorial Biosynthesis and Drug Discovery, Ministry of Education, School of Pharmaceutical Sciences, Wuhan University, Wuhan, China

**Keywords:** single-cell sequencing, FMDV, persistent infection, MAPK signaling pathway, Fos

## Abstract

Foot-and-mouth disease virus (FMDV) could cause acute infection in host cells, or they could coexist with host cells to generate persistent infection. In persistent infection, the virus could survive for a long time in the host and could be transmitted between different host cells. In the case of FMDV-persistent infection cell line, there is a remarkable significant cellular heterogeneity in the FMDV-persistent infection cell line due to differences of viral load in the individual cells within the cell line. However, the mechanisms of FMDV-persistent infection are not well understood. It is now generally accepted that multiple factors contribute to the coevolution of viruses and cells during the course of persistent infection. The outcome would influence the development of persistent FMDV infection conjointly, reaching a state of equilibrium ultimately. Therefore, in order to elucidate the mechanism of cellular heterogeneity in FMDV-persistent infection cell line, single-cell sequencing was performed on BHK-Op, and pseudotime trajectory plot was draw through cell cluster. Based on the cell clusters, we predicted the development and progression of the FMDV-persistent infection. It could be well explained by the fact that, in BHK-Op cells, there are a fraction of infected cells and a fraction of virus-exposed but uninfected bystander cells. By further comparing the transcripts in cell clusters, we found that these genes were involved in changes in ribosome biogenesis, cell cycle, and intracellular signaling including the interferon signaling pathway and mitogen-activated protein kinase (MAPK) signaling pathway. Through comprehensive cross-tabulation analysis of differential expressed genes in various cluster of cells, we identified a high association of Fos, a downstream transcription factor of the MAPK/extracellular signal–regulated kinase (ERK) signaling pathway, with viral replication during the formation of FMDV-persistent infection. Through the further study of Fos, we found that downregulation of Fos facilitates viral clearance during FMDV-persistent infection. Upregulation of c-Raf, which is the upstream of the MAPK/ERK signaling pathway, could promote FMDV replication through downregulation of Fos. Our research is the first to provide insight into the mechanism of the formation FMDV-persistent infection through single-cell sequencing using persistent infection cell line. Pseudotime trajectory analysis was the first time to apply for FMDV-persistent infection cell line. Our work highlights the detailed overview of the evolution of FMDV-persistent infection. We also analyzed the differential expressed genes in the replication or elimination of FMDV within the host. We found that the MAPK/ERK signaling pathway and its downstream transcription factor Fos play an important role in FMDV-persistent infection.

## Introduction

Foot-and-mouth disease (FMD) is a highly infectious and harmful disease that infects cloven-hoofed animals and could easily cause large-scale outbreaks, with effects such as sloping feet, low productivity, and reduced milk production, which could cause serious economic losses in epidemic areas (James and Rushton, 2002). The pathogen, FMD virus (FMDV), belongs to the family *Picornaviridae*, which is a single- and positive-stranded RNA virus, that has been classified serologically into seven serotypes (A, O, C, Asia 1, and South African Territories 1, 2, and 3) (Salem et al., 2019). The wide host range of FMDV and the presence of persistent infection make it difficult to control once it spreads (Pattnaik et al., 2012).

Under natural conditions, the infection of FMDV in host cells could cause a cytopathic effect (CPE), resulting in acute infection (Qian et al., 2015). However, it could also cause persistent infection through mildly coexisting with host cells (De La Torre et al., 1988; Domingo et al., 2003). In our previous studies, we have successfully established a model of FMDV-persistent infection cells BHK-Op and bystander cells BHK-VEC (virus-eliminated cell). Using NH_4_Cl treatment and single-cell isolation technique, we obtained the BHK-21 cells which FMDV could survive in the host cells for a long time after infection and could not produce violent humoral reactions, or release virulent particles continuously (Huang et al., 2009; Huang et al., 2011). This cell line was named BHK-Op, and the pathogenic virus was named FMDV-Op. The virus negative cell line isolated from the persistent infection cell line by gradient dilution was named BHK-VEC (Han et al., 2018). Our previous research also revealed that BHK-VEC was resistant to wild-type FMDV, but the infection of BHK-VEC with FMDV-Op could reacquire FMDV-persistent infection. The establishment of the persistent infection cell line is of great significance for the further understanding of the relationship between FMDV and host (Huang et al., 2011).

There is significant heterogeneity in the process of viral infection. The presence of defective interfering particles or viral variant, host cell heterogeneity, and gene regulation is mainly reflected in the transcriptional level (Andreu-Moreno and Sanjuán, 2020). Therefore, transcriptome information is of great importance for the study of the viral infection process. And with the development of single-cell isolation techniques from micromanipulation to machine sorting such as fluorescence-activated cell sorting, the study of cellular heterogeneity of the viral infection process is more convenient to be carried out at the single-cell level (Zhang et al., 2013; Xin et al., 2018). Single-cell sequencing is the analysis of a single cell at a specific time and in a specific state, obtaining a large amount of transcript information of the cell (Shapiro et al., 2013). Single-cell sequencing could exclude the interference of multicell population which has a positive effect on the study of the host of viral infection. In the earlier analysis of the single-cell sequencing, PCR technology was applied to amplify cDNA from individual cells, but it was limited that, in the analysis, there were only a few hundred genes (Tang et al., 2009). Later, the expanded use of DNA microarrays enabled the development of single-cell microarray technology, whose advantages of high throughput were overwhelming (Bumgarner, 2013). Although this method has gained great progress in obtaining whole gene expression forms, it did not detect changes in alternative splicing in transcriptome information, and unknown genes could not be detected through this method. The single-cell sequencing technology is a landmark advancement that brings together the advantages of high throughput and whole gene to provide new perspectives on transcriptome information (Bankevich et al., 2012). In this research, single-cell sequencing was used to analyze the transcripts of FMDV-persistent infection cell line BHK-Op, in order to obtain the expression of cellular genes in different infection states and then use bioinformatics analysis to further explain the mechanism of FMDV-persistent infection in the host cells.

Our previous experiments demonstrated that the rate and virulence of infection of BHK-21 by the persistent infection virus FMDV-Op is almost indistinguishable from that of FMDV, which excluded viral mutations or defects in the formation of persistent infection cells (Han et al., 2018). And we have known that persistent infection cells are resistant to wild-type FMDV and that viral immune escape factors in host cells might promote the formation of persistent infection cells (Wang et al., 2020). MAPK/p38 and MAPK/ERK-related signaling pathways were active in the late phase of acute infection, and factors associated with viral replication are differentially expressed in persistent infection cells, such that persistent infection cells achieve their resistance to viral replication by downregulating the expression of MAPK14 and Hspb1 in the MAPK signaling pathway (Li et al., 2020). Emopamil Binding Protein (EBP) protein was shown to promote FMDV replication, and EBP expression was upregulated in acutely infected BHK-21 cells, whereas the opposite was true in persistently infected cells (Fang et al., 2017). Our experiments have also verified that Cav1 was required for FMDV-Op proliferation during persistent infection, and that downregulation of Ccnd1 was one of the causes of wild-type FMDV resistance in BHK-Op cells (Han et al., 2021).

## Materials and Methods

### Library Constructing and Single-Cell Sequencing

After completing the library creation, the library was quantified using Qubit2.0 Fluorometer and diluted to 1.5 ng/μl. The insert size of the library was then examined using Agilent 2100/4200 bioanalyzer until it met expectations. RT-qPCR was performed to accurately quantify the effective library concentration (effective library concentration above 2 nM) to ensure library quality. The different libraries that pass the test were pooled according to the effective concentration and the target output data volume then subjected to Illumina sequencing. It was based on the principle of sequencing by synthesis, in which fluorescently labeled dNTP, DNA polymerase, and splice primers were added to the sequencing flow cells for amplification. When each sequencing cluster extended the complementary strand, each addition of fluorescently labeled dNTP released a corresponding fluorescence, and the sequencer obtained the information of the fragment to be sequenced by capturing the fluorescence signal and converting the light signal into sequencing peaks.

### Pseudotime Trajectory and Functional Enrichment Analysis

Single-cell sequencing libraries were used for quantitative analysis of cellular gene expression, data quality and control analysis, comparison analysis, gene expression quantification and saturation analysis, cellular gene expression Quality control (QC), cellular subgroup clustering, cluster average UMI calculation, cluster correlation analysis, cellular typing downscaling display [T-distributed stochastic neighbor embedding (tSNE)/Uniform Manifold Approximation and Projection (UMAP)], cluster differential gene analysis, Monocle2 trajectory analysis, simulated timeline analysis, marker gene display, enrichment analysis [Gene Ontology (GO) enrichment, reactome enrichment, Kyoto Encyclopedia of Genes and Genomes (KEGG) pathway enrichment], protein interaction network analysis, and differential gene transcription factor annotation. In the cellular gene expression QC, the number of genes in cells (nFeature), the number of UMIs in cells (nCount), the distribution ratio of mitochondrial gene content in cells (per cent.mito), and the expression ratio of genes encoding ribosomal proteins in cells (per cent.Ribo) were mapped. Then, the abnormal cells were removed before subsequent analysis. The nFeature, nCount, and percent.mito, and the expression ratio of genes encoding ribosomal proteins in the cells (percent.Ribo) were first analyzed. These indicators were analyzed in a descending manner for the cell. After sorting the samples into individual cell subtypes, the mean unique molecular identifier (UMI) value of each gene could be obtained for each cluster within the sample, and further analysis could be performed based on this. For a large number of single-cell samples, the distribution state of the cells needs to be displayed by dimensionality reduction operation; the analysis process uses PCA (principal components analysis), tSNE, and UMAP for dimensionality reduction display. Single-cell data facilitate the study of cell dynamics, especially cell cycle, cell differentiation, and cell activation, and these dynamic processes could be trajectory analyzed using Monocle2 software based on previous cluster classification results. Seurat software provides FindAllMarkers module to identify marker gene of all clusters, and the results of marker genes identified by this software are presented in this section for graphical analysis.

### Cells and Viruses

BHK-21 was obtained from China Center for Type Culture Collection. FMDV type O was provided by Lanzhou Veterinary Research Institute, Chinese Academy of Agricultural Sciences. FMDV-persistent infection cells BHK-Op and bystander cells BHK-VEC were collected from our laboratory. Briefly, the BHK-21 cells were infected with FMDV and treated with 20 mM NH_4_Cl. Then, single cells were cultured and passaged through isolating by microscopic manipulation to establish the persistent infection cell line (BHK-Op). During the BHK-Op passaging process, the RNA levels of FMDV in the cells at each generation were detected. Thereby, we identified a virus-negative cell line in BHK-Op. It was named BHK-VEC.

The above cell line was cultured at 37°C in 5% CO_2_ incubator in minimum essential medium (MEM) (Gibco, USA) with 10% fetal bovine serum (FBS) (Excell, China). The incubated cells were allowed to grow to about 95% confluence, removed the culture supernatant and added trypsin to completely separate cells, neutralized with MEM, centrifuged the cell suspension, and added fresh medium with 10% FBS. And the culture volume was expanded for the purpose of expanding the cell culture. Because BHK-Op cells continue to be toxic, the cells need to be replaced with fresh medium at 24-h intervals, and we usually gave passaging treatment when BHK-Op proliferation reaches 70% confluence. Approximately, 4.5 lgTCID50 of FMDV was used in the routine virus infection procedure. In case of infection, the cell supernatant needs to be discarded and washed once with PBS. After adding the virus, the cells should be incubated at 37°C for 1 h, followed by discarding the virus and replacing it with MEM medium containing FBS.

### RNA Extraction and RT-qPCR

The cells in the well plate were treated at a specific time, removed the culture supernatant, washed three times with PBS, and TRIzol reagent (Life technologies, USA) was used for RNA extraction. Briefly, lysed by adding appropriate amount of TRIzol to release RNA, then added chloroform and centrifuged to collect the aqueous phase, rinsed with isopropanol and 75% ethanol, and finally dried and dissolved to obtain cellular RNA, and the sample concentration was determined by NanoDrop 2000 and placed in −80°C refrigerator for use. RNA samples were reverse transcribed to cDNA by MMLV (Promega, USA) before being measured by RT-qPCR. cDNA and Taq Pro Universal SYBR qPCR Master Mix (Vazyme, China) were added in a 96-well plate and detected. Primers used in the experiment were listed in **Table 1**. The expression level of glyceraldehyde-3-phosphate dehydrogenase (GAPDH) was used as an internal reference, and the relative changes of target genes were calculated by the 2^-△△CT^ method.

**Table 1:** Primers sequences used in this research.

Primer	Sequence (5’-3’)
FMDV 3D-FP	GAACACATTCTTTACACCAGGAT
FMDV 3D-RP	CATATCTTTGCCAATCAACATCAG
C-Raf-FP	GGATTTCGGTGTCAGACTTG
C-Raf-RP	CATTGGGAGTGGATGTTGAC
FOS-FP	CAGATCTGTCCGTCTCCAGT
FOS-RP	GACACGGTCTTCACCATTCC
GAPDH-FP	AAGGCCATCACCATCTTCCA
GAPDH-RP	GCCAGTAGACTCCACAACATAC

### Gene Knockdown by Short-Hairpin RNA

The short-hairpin RNA (shRNA) was designed according to the cDNA sequence of the target gene, and the sequences of shRNA were listed in **Table S1**. The strands of the oligos that contain the shRNA were annealed. pSUPER.retro vector was linearized *via BglII* and *HindIII* (Thermo Fisher Scientific, USA). The annealed strands of the oligos were cloned into the vector, and then transformed into *E. coli* DH5α.

### Cell Transfection

The cells were cultured in 12-well plates and transfected when grew to 80% confluence, and the transfection solution Lipofectamine 2000 (Thermo Fisher Scientific, USA) was a mixture of liquid A and liquid B; liquid A was a mixture of 200 μl of Opti-MEM (Thermo Fisher Scientific, USA) containing 2.4 μg plasmids, and liquid B was a mixture of 200 μl of Opti-MEM containing 2.4 μl of Lipofectamine 2000. Liquid A and liquid B were configured to stand for 5 min, and then mixed, and placed at room temperature for 30 min to form the complex of liposome and plasmid. The cell culture medium was discarded and washed with serum-free MEM. Then, the transfection mixture was transferred into it, after 4–6 h, changed to MEM medium containing 2% FBS, and placed in the incubator to continue the culture for subsequent operation.

### Western Blot

The cells were lysed by SDS loading buffer containing radioimmunoprecipitation assay lysate (Beyotine, China). The protein lysate was denatured at 95°C and centrifuged, and then added the supernatant to the configured SDS–polyacrylamide gel electrophoresis gel for protein separation. The gel was then transferred to methanol-activated polyvinylidene difluoride (PVDF) membrane (Millipore, USA), and the membrane was washed by Tris Buffered Saline with Tween (TBST) (Coolaber, China) and blocked for 30 min by 5% skim milk at room temperature. The membrane was washed with TBST three times for 5 min each time and incubated overnight at 4°C with primary antibody, and the antibodies used in the experiment were listed in **Table 2**. The membrane was washed three times with TBST, added corresponding horseradish peroxidase (HRP)-labeled secondary antibody, incubated for 1–2 h at 37°C. The membrane was washed with TBST three times for 5 min each time and incubated the PVDF membrane in the ECL solution (Millipore, USA) for 1-2 min, and development was performed with the ChemiDoc XR system (Bio-Rad, USA).

**Table 2:** Antibodies used in this research.

Antibody	Description	Multiple of dilution
FMDV 3D Rabbit pAb	Manufactured by Abiocenter (China)	1:2000
FOS Rabbit pAb	Abclonal (China) A0236	1:1500
Anti-ERK1/2 Recombinant Rabbit Monoclonal Antibody	Huabio (China) ET1601-29	1:4000
Anti-Erk1 (pT202/pY204) + Erk2 (pT185/pY187) Recombinant Rabbit Monoclonal Antibody	Huabio (China) ET1610-13	1:3000
Anti-p38 Recombinant Rabbit Monoclonal Antibody	Huabio (China) ET1602-26	1:3000
Anti-Phospho-P38 (Thr180 + Tyr182) Rabbit Polyclonal Antibody	Huabio (China) ER2001-52	1:3000
Anti-JNK1+JNK2+JNK3 Recombinant Rabbit Monoclonal Antibody	Huabio (China) ET1601-28	1:2000
Anti-Phospho-JNK1/2/3(T183+T183+T221) Recombinant Rabbit Monoclonal Antibody	Huabio (China) ET1609-42	1:2000
GAPDH Mouse mAb	Abclonal (China) AC033	1:5000
HRP Goat Anti-Rabbit IgG (H+L)	Abclonal (China) AS014	1:5000
HRP Goat Anti-Mouse IgG (H+L)	Abclonal (China) AS003	1:5000

### Tissue Culture Infective Dose Assay

Tissue culture infective dose (TCID_50_) assay was performed to determine the titer of FMDV. Briefly, BHK-21 or BHK-VEC cells were inoculated into 96-well plates at the amount of 2 × 10^4^ per well uniformly, incubated in a 37°C incubator with 5% CO_2_. When the cells confluence reached 70%, the old medium was aspirated and discarded, and washed twice with PBS. Virus was diluted by 10-fold gradient with MEM medium and 100 μl of it was added. Serum-free medium was used as a negative control and there were eight replicate wells per dilution. The cells were incubated for 1 h, aspirated, and discarded the virus solution, and MEM containing 2% FBS was added and incubated for 72 h. The occurrence of CPE in each well was then observed and recorded with an inverted microscope. The titer of FMDV was calculated according to Reed-Muench.

### Statistical Analysis

Data were expressed as the means ± standard deviation. All statistical analyses were performed with variance using SPSS version 16.0 software (SPSS, Osaka, Japan). A value of *p* < 0.05 was considered to indicate a statistically significant difference.

## Results

### Comprehensive FMDV-Persistent Infection Map by Single-Cell Sequencing

**Figure 1:**
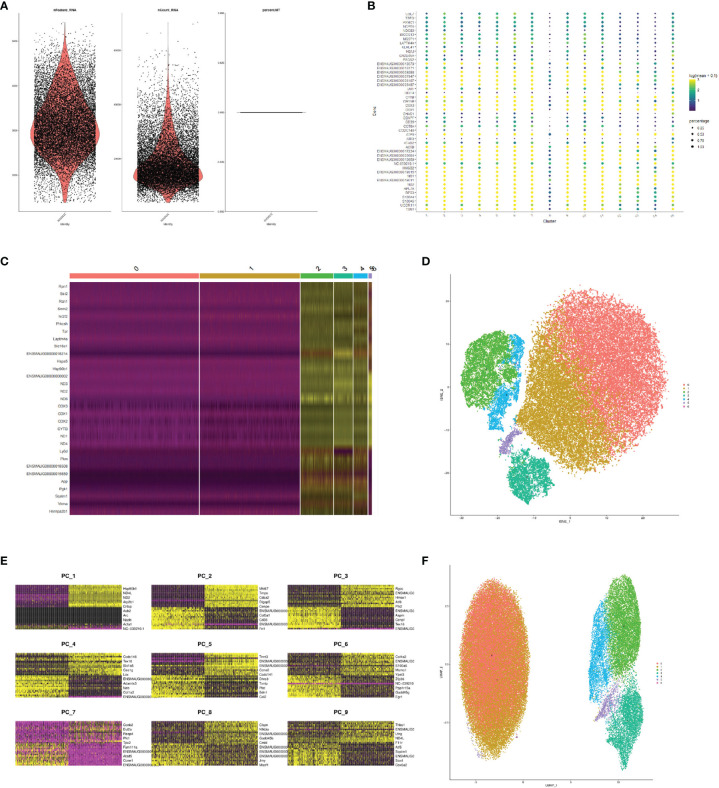
Comprehensive FMDV-persistent infection map by single-cell sequencing. **(A)** Violin diagram of the quality control of single-cell sequencing. Dot diagram **(B)** and the heat map **(C)** of the gene clustering of cell subpopulations. tSNE displayed subclusters after cluster analysis **(D)**. Heat map of the genes in the first nine principal components **(E)**. UMAP displayed subclusters after cluster analysis **(F)**.

In this study, the FMDV-persistent infection cell line BHK-Op of 33 passages was collected for single-cell sequencing. The estimated number of cells was 17,551. The mean reads per cell were 59,181. The median genes per cell were 2,977. There were 96.9% valid barcodes and 100% valid UMIs in the sequencing. The fraction reads in cells were 90.6%. A total of 14,057 genes were detected. And the median UMI counts per cell were 15,675. After obtaining the sample cell expression matrix, some cells could have abnormal mitochondrial gene content or UMI numbers due to the presence of low cell activity, doublets during sample processing. Therefore, we first examined the number of genes in the cell (nFeature), the number of UMIs in the cell (nCount), and the proportion of mitochondrial gene content in the cell (per cent.mito) distribution. The results of nFeature, nCount, and percent.mito in our cells were shown in **Figure 1A**. Our samples were derived from the same strain of cells; thus, the same number of mitochondria was shown in our results. Using the gene clustering bubble plots of cell subgroups (**Figure 1B**), we adjusted the subsequent more desirable clustering results to a total of seven cell clusters. Furthermore, the gene clustering heat map of cell subgroups was used to confirm the comparison to find the genes that might be characteristic of the subgroups (**Figure 1C**). Following the clustering analysis, we presented the results of the tSNE descending binning of our sample (**Figure 1D**). Each scatter represents a cell in the diagram, resulting in a total of seven cell clusters. In order to cluster the cells by UMAP, we first clustered the genes in the first nine principal components (**Figure 1E**). The UMAP clustering display after the clustering analysis resulted inthe same division of the sample into seven cell clusters (**Figure 1F**).

### Reconstruction of Persistent Infecting Trajectory Through Pseudotime Analysis

**Figure 2:**
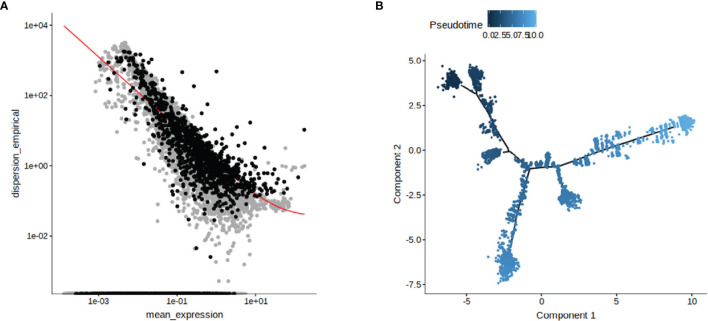
Reconstruction of persistent infecting trajectory through pseudotime analysis. The genes used for the proposed pseudotime trajectory analysis after screening were identified **(A)**. DDRTree method was used to show the distribution of pseudotime trajectory in two dimensions **(B)**. The developmental trajectory of the cells is distinguished by the shade of color.

The data of single-cell sequencing facilitated the study of cell dynamics, particularly cell cycle, cell differentiation, and cell activation. And these dynamic processes could be computationally simulated using trajectory information methods. We performed a trajectory analysis using Monocle2 software based on the previous cluster classification results. Genes were screened for subsequent use in pseudotime trajectory analysis based on the average expression between cells showing differences in genes (**Figure 2A**). The DDRTree method was used to downscale the distribution of cell pseudotime in two dimensions, showing the developmental trajectory of cells from deep to shallow (**Figure 2B**). It was clear from our results that the FMDV-persistent infection cell BHK-Op was differentiated from one cell cluster to two different clusters, which explained the presence of two different types of cells in BHK-Op cells, the infective cells that persistently carry the virus, and the bystander cells that have cleared the virus.

### Characterization of the Functional Enrichment Response During FMDV-Persistent Infection

**Figure 3:**
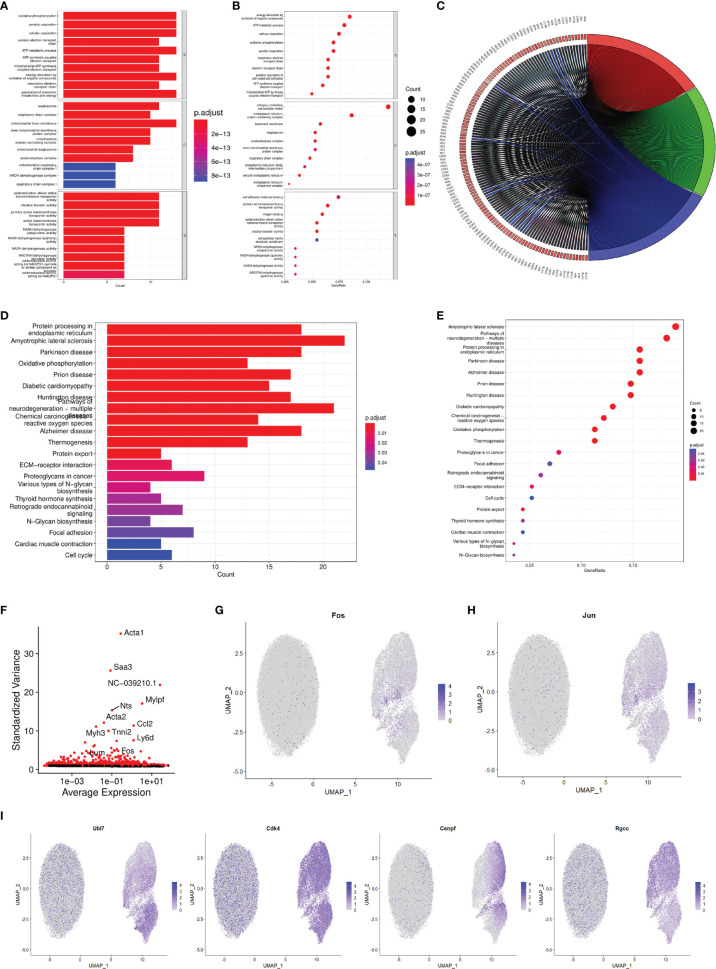
Characterization of the functional enrichment response during FMDV-persistent infection. Bar **(A)** and dot **(B)** diagram of the GO enrichment of BHK-Op. The dot from small to big corresponds to a small to large amounts of gene expression, and the color from blue to red corresponds to a low to high abundance of gene expression. Chord diagram **(C)** showed the relationship between genes and individual GO items. Bar **(D)** and dot **(E)** diagram of the KEGG enrichment of BHK-Op. The dot from small to big corresponds to a small to large amounts of gene expression, and the color from blue to red corresponds to a low to high abundance of gene expression. Scatterplot of gene dispersion **(F)** labeled the top fifteen genes with the greatest amount of variation. In two dimensions, the expression levels of FOS **(G)**, JUN **(H)**, and four other randomly selected genes **(I)** were displayed. The color light to dark corresponds to a low to high abundance of gene expression.

Based on the data of single-cell sequencing, we performed GO enrichment analysis on the individual BHK-Op cell clusters. The results showed that, in terms of biological process, energy derivation by oxidation of organic compounds, ATP metabolic process, and cellular respiration were enriched. In terms of cellular components, collagen-containing extracellular matrix, endoplasmic reticulum protein-containing complex, and basement membrane were enriched. In terms of molecular function, cell adhesion molecule binding, primary active transmembrane transpoter activity, and integrin binding were enriched (**Figures 3A, B**). The overall presentation of different genes in different GO-enriched pathways on BHK-Op cells was displayed in chord diagram (**Figure 3C**). Furthermore, we performed KEGG enrichment analysis on the individual cell clusters of BHK-Op, and the results of KEGG enrichment analysis of BHK-Op showed that protein processing in endoplasmic reticulum, amyotrophic lateral sclerosis, oxidative phosphorylation was significantly enriched (**Figures 3D, E**). Of all the differentially expressed genes, we screened the top 5,000 and annotated the top 15 with the greatest amount of gene variation. As shown in **Figure 3F**, the vertical coordinates are the outlier values of the genes, with larger outlier values indicating greater gene variability. These include the MAPK signaling pathway downstream transcription factor FOS. Therefore, we showed the expression levels of several genes of our interest in all cells by downscaling, in two dimensions, the transcription factors downstream of the MAPK signaling pathway, for FOS as in **Figure 3G** and for Jun as in **Figure 3H**. And several additional genes were randomly selected, and we also displayed their expression levels in all cells by downscaling (**Figure 3I**). It is noted that there were multiple signaling pathways that are differentially expressed in the individual cellular clusters of BHK-Op, all of which were highly likely to regulate the development of persistent FMDV infection.

### MAPK Signaling Pathway Was Activated Under the Infection of FMDV With Reliance on Viral Replication

**Figure 4:**
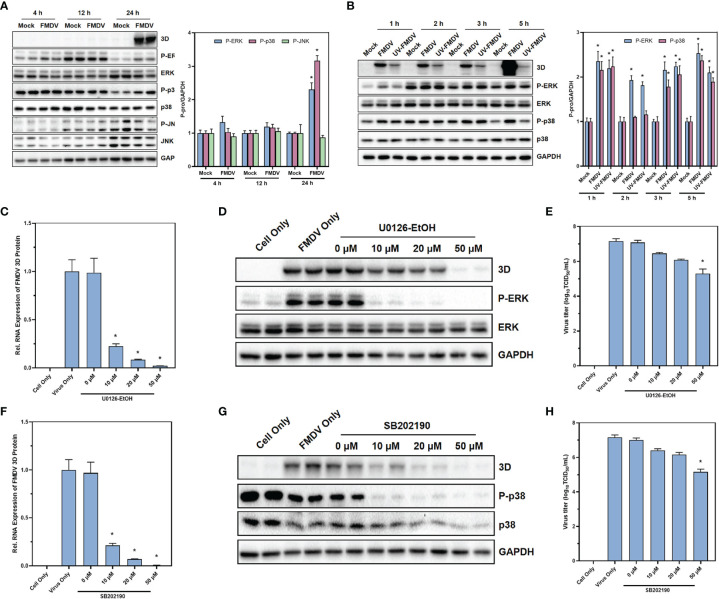
MAPK signaling pathway was activated under the infection of FMDV with reliance on viral replication. The protein expression of the marker proteins in MAPK signaling pathway after the infection of FMDV **(A)** or UV-treated FMDV **(B)** was confirmed by Western blot. The effects of MAPK/ERK signaling pathway inhibitor U0126-EtOH-EtOH on RNA levels **(C)**, protein levels **(D)**, and infectious FMDV virus titers **(E)** and the effects of MAPK/p38 signaling pathway inhibitor SB202190 on RNA levels **(F)**, protein levels **(G)**, and infectious FMDV virus titers **(H)**. The intensity of pERK, p38, and p-JNK relative to GAPDH was measured by ImageLab software. GAPDH was served as the internal reference to normalize data. Three independent replicates were conducted for each sample. Data are expressed as the means ± SE (n = 3). *P < 0.01.

Based on the results of single-cell sequencing, the results of GO enrichment analysis and KEGG enrichment analysis, as well as the analysis of the expression of individual transcription factors, we found that the MAPK signaling pathway is intimately associated with FMDV infection. Therefore, to verify the effect of MAPK signaling pathway on FMDV, we first examined the marker proteins of MAPK signaling pathway and their phosphorylation levels after 4, 12, and 24 h of FMDV infection. We found that the phosphorylation levels of ERK and p38 proteins were significantly increased at 24 h of FMDV infection (**Figure 4A**). To verify the effect of FMDV replication on the MAPK signaling pathway, the approximately 2.5 lgTCID_50_ UV-inactivated FMDV was used to infect BHK-21 cells and showed that UV-inactivated FMDV did not significantly alter the phosphorylation levels of key proteins of MAPK signaling pathway (**Figure 4B**). In this manner, we treated BHK-21 cells with different concentrations of U0126-EtOH, a specific inhibitor of the MAPK/ERK signaling pathway, and SB202190, a specific inhibitor of the MAPK/p38 signaling pathway, respectively. The concentrations of U0126-EtOH and SB202190 used in our experiments were not of significant toxicity to the cells (**Figures S1**, **S2**). After the treatment of U0126-EtOH-EtOH (**Figures 4C–E**) and SB202190 (**Figures 4F–H**), subsequent to FMDV infection, we found that RNA levels, protein levels, and infectious virus titers were significantly reduced in FMDV. Our results showed that specific modulation of both the MAPK/ERK signaling pathway and the MAPK/p38 signaling pathway significantly inhibits the replication of FMDV and the ability of FMDV to become infected. Furthermore, the effects of MAPK/ERK and MAPK/p38 signaling pathways on FMDV were dependent on the replication of FMDV itself.

### Contributions of MAPK/ERK Signaling Pathway in Acute and Persistent FMDV Infection

**Figure 5:**
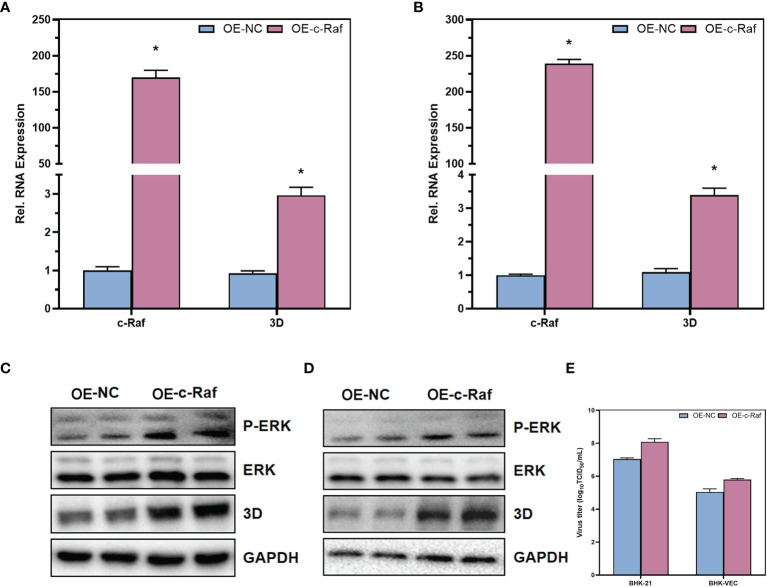
Contributions of MAPK/ERK signaling pathway in acute and persistent FMDV infection. The RNA expression levels of c-Raf and FMDV 3D in BHK-21 cells **(A)** and BHK-VEC cells **(B)** were verified by RT-qPCR. The protein expression levels of ERK and FMDV 3D and the phosphorylation of ERK in BHK-21 cells **(C)** and BHK-VEC cells **(D)** were verified by Western blot. The infectious FMDV titers in BHK-21 cells and BHK-VEC cells **(E)** were verified by TCID_50_ assay. OE, over expression; NC, negative control. GAPDH was served as the internal reference to normalize data. Three independent replicates were conducted for each sample. Data are expressed as the means ± SE (n = 3). *P < 0.01.

With U0126-EtOH-EtOH, a specific inhibitor of the MAPK/ERK signaling pathway, we found that inhibition of the MAPK/ERK signaling pathway significantly suppressed RNA levels, protein levels, and infectious virus titers in FMDV. Therefore, to further validate the role of MAPK/ERK signaling pathway on FMDV, we overexpressed c-Raf, a key kinase of the MAPK/ERK signaling pathway, in BHK-21 and BHK-VEC cells. The c-Raf kinase is commonly highly expressed in all types of tumor cells to facilitate higher activity of the MAPK signaling pathway. Our experiments simulated acute infection of FMDV by infecting BHK-21 cells with wild-type FMDV. Persistent infection of BHK-VEC cells by persistent FMDV virus mimicked persistent infection by FMDV. Therefore, we overexpressed c-Raf in BHK-21 and BHK-VEC, constructed c-Raf high-expressing BHK-21 and BHK-VEC cells, and infected BHK-21 and BHK-VEC cells with FMDV virus and FMDV virus produced by sense-holding cells, respectively. The RNA levels of the three-dimensional (3D) protein of FMDV were measured by RT-qPCR and showed that the RNA levels of the FMDV 3D protein were significantly upregulated in both BHK-21 (**Figure 5A**) and BHK-VEC (**Figure 5B**) cells after the overexpression of c-Raf. The phosphorylation levels of ERK kinase downstream of c-Raf were also examined by Western blot. The results showed that the phosphorylation levels of ERK were significantly increased after high expression of c-Raf, and the protein levels of 3D of FMDV were also significantly upregulated in BHK-21 (**Figure 5C**) and BHK-VEC (**Figure 5D**) cells. Through TCID_50_ assays, the results showed that overexpression of c-Raf also contributed to infectious FMDV viral titers (**Figure 5E**). Our results revealed that activation of MAPK/ERK signaling pathway could promote acute and persistent FMDV infection significantly.

### The Effects of Transcription Factor FOS on Acute and Persistent FMDV Infection

**Figure 6:**
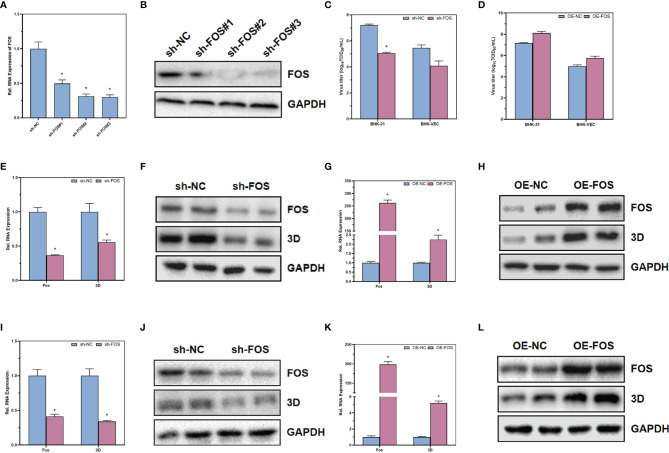
The effects of transcription factor FOS on acute and persistent FMDV infection. The RNA and protein expression levels of FOS in BHK-21 after transfected with shRNA were verified by RT-qPCR **(A)** and Western blot **(B)**. The infectious FMDV titers in BHK-21 cells **(C)** and BHK-VEC cells **(D)** were verified by TCID_50_ assay. The RNA and protein expression levels of FOS and FMDV 3D in BHK-21 after downregulation of FOS in BHK-21 cells were verified by RT-qPCR **(E)** and Western blot **(F)**. The RNA and protein expression levels of FOS and FMDV 3D in BHK-21 after upregulation of FOS in BHK-21 cells were verified by RT-qPCR **(G)** and Western blot **(H)**. The RNA and protein expression levels of FOS and FMDV 3D in BHK-VEC after downregulation of FOS in BHK-VEC cells were verified by RT-qPCR **(I)** and Western blot **(J)**. The RNA and protein expression levels of FOS and FMDV 3D in BHK-VEC after upregulation of FOS in BHK-VEC cells were verified by RT-qPCR **(K)** and Western blot **(L)**. Sh, shRNA; OE, over expression; NC, negative control. GAPDH was served as the internal reference to normalize data. Three independent replicates were conducted for each sample. Data are expressed as the means ± SE (n = 3). *P < 0.01.

Through the analysis of differential genes in single-cell sequencing, we found that FOS, a transcription factor downstream of the MAPK/ERK signaling pathway, was differentially expressed between cells of different clusters. To further validate that the MAPK/ERK signaling pathway could regulate acute and persistent infection in FMDV *via* FOS, shRNA was designed to knock down the expression of FOS. Through RT-qPCR (**Figure 6A**) and Western blot (**Figure 6B**), we verified that FOS was knocked down successfully. In BHK-21, FOS was knocked down and infected with wild-type FMDV to construct a cellular model of acute FMDV infection. We found that knockdown of FOS significantly inhibited both RNA levels and protein levels of FMDV 3D protein by RT-qPCR (**Figure 6E**) and Western blot (**Figure 6F**). In contrast, upregulation of FOS expression in BHK-21 cells significantly promoted the RNA levels and protein levels of FMDV 3D protein (**Figures 6G, H**). We constructed a model of persistent infection of FMDV by infecting BHK-VEC cells with FMDV-Op. The results showed that knocking down FOS in BHK-VEC similarly suppressed the RNA level and protein level of FMDV 3D protein (**Figures 6I, J**). In contrast, upregulation of FOS expression in BHK-VEC cells also significantly promoted the RNA level and protein level of FMDV 3D protein (**Figures 6K, L**). Downregulation of FOS in BHK-21 cells and BHK-VEC cells could significantly inhibit the titers of infectious virus by TCID_50_ assay (**Figure 6C**). Upregulation the expression of FOS could promote the titers of infectious FMDV virus (**Figure 6D**). Our results suggested that FOS could facilitate FMDV replication and enhance FMDV infectivity, both in acute and persistent FMDV infections.

## Discussion

Single-cell sequencing is a technology created in recent years in the field of biology. Due to its great resolution, single-cell sequencing approaches to precisely profile information about a sample’s cellular composition (Kolodziejczyk et al., 2015). There is a lot of promise for a wide range of applications with the advancement of single-cell sequencing technology. Overall, single-cell sequencing is currently being used to build global cellular landscapes and identify disease identity markers. Many applications of single-cell sequencing in the tumor microenvironment as well as the immune microenvironment have been documented in recent studies (Gu et al., 2021; Wang et al., 2021b). Because tumors are extremely diverse, this diversity has a significant impact on disease development and therapeutic efficacy (Vasudev and Reynolds, 2014). Knowledge in the tumor microenvironment and immune microenvironment allows us to obtain a better understanding of the tumor and, as a result, to find prospective therapeutic targets (Guo et al., 2018). In the study of the tumor microenvironment, single-cell sequencing is becoming routinely used (Lavin et al., 2017). Furthermore, the study found that by mapping single cells in the gastric mucosa of patients with nonatrophic gastritis, atrophic gastritis, and early gastric cancer, the changes in epithelial cells during the process from gastritis to gastric cancer revealed a gradual decrease in the percentage contribution of highly differentiated cell types in the stomach and the appearance of stem cell–like cells during the period of intestinal epithelial metaplasia, reaching the higher level of intestinal (Zilionis et al., 2019). Single-cell sequencing could possibly be employed in cancer therapy to highlight evolutionary mechanisms. Single-cell sequencing was performed on tumor samples before targeted therapy, tumor samples that remained after targeted therapy response, and tumor samples that had relapsed after targeted therapy. Possible resistance pathways were identified by comparing the features of samples from different eras, exposing the mechanisms of resistance to targeted therapy in lung cancer and suggesting potential therapeutic targets (Maynard et al., 2020).

In addition to its applications in the field of research, single-cell sequencing has also become increasingly useful as a clinical guide. Single-cell sequencing could improve therapeutic outcomes through guiding the clinical use of drugs. Kim et al. reported a special patient who developed a severe and life-threatening drug allergy syndrome while undergoing targeted drug therapy. Single-cell sequencing was used to compare the skin samples from this patient with those from healthy people. The JAK/STAT pathway was identified as a potential target in the skin samples of this patient. After treatment with tofacitinib, the skin inflammation was eliminated and the life of the patient was saved (Kim et al., 2020). Single-cell sequencing is also beginning to be investigated in immunotherapy plus chemotherapy for patients with epidermal growth factor receptor mutations after targeted therapy failure (Wang et al., 2021a). Moreover, single-cell sequencing could be used for target development and extended drug applications. In recent research, single-cell sequencing was performed on melanoma patient samples before and after immunotherapy, and the treated patients were divided into drug-resistant and non–drug-resistant groups. The comparison revealed that in the drug-resistant patients, T-cell infiltration became lower and the CDK4/6 signaling pathway was abnormally active in the drug-resistant group. CDK4/6 inhibitors are currently clinically approved for the treatment of human epidermal growth factor receptor-2 positive breast cancer, which are also being used in a variety of other cancers (Jerby-Arnon et al., 2018). Single-cell sequencing has proved to be of great advantage in the discovery of new drug targets, new approaches to old drugs and immunotherapeutic combinations, and will later provide a strong theoretical basis for the clinical treatment of various diseases. Recent research has demonstrated that single-cell sequencing could be applied to the precise detection of pathology. Single-cell sequencing was used to study patients with azoospermia, resulting in the identification of previously unreported macrophages, myxoid cells, and mesenchymal cells in testicular samples from nonobstructive azoospermia. Only somatic cells were present in the NOA.1 sample from the results, which might manifest as a support cell only syndrome. This study suggests that single-cell sequencing might be as useful as histopathology in classifying NOA at the level of precise cell based compartmentalization (Chen et al., 2020).

It is the first time that single-cell sequencing was applied for giving insights into the mechanism of the formation of BHK-Op, which is a FMDV-persistent infection cell line obtained from our laboratory earlier. Structural features in high-dimensional space were projected into 2D and 3D space by tSNE and UMAP, using the sparsity and remoteness of points in planar or 3D space to represent their sparsity and remoteness in an otherwise multidimensional state. We divided the BHK-Op cells into a total of seven different clusters according to different markers. Due to the properties of BHK-Op cells, it is formed by FMDV infection of BHK-21 cells. In our previous studies of BHK-Op cells, we found that, in BHK-Op cells, on the one hand, FMDV could coexist with cells. On the other hand, FMDV was eliminated from cells that did not contain any virus. Our tSNE and UMAP fractionation results similarly showed that there were two distinct cell populations in BHK-Op cells. Based on the results of single-cell sequencing, we analyzed the pseudotime trajectory of the cells in the individual clusters. Our study is the first to describe the changes in cell differentiation and cell activation during the formation of FMDV-persistent infection at the cellular level using single-cell sequencing. However, there are still some problems that remain to be solved, and one of them is that, due to species differences, we failed to annotate the FMDV viral genome in the results of single-cell sequencing. If this problem could be solved, we could track the RNA levels of FMDV in each cell cluster, which would present a more intuitive picture of the evolution of FMDV-persistent infection. GO enrichment analysis of cells from all clusters showed that differential genes in BHK-Op cells were enriched in the cellular processes of oxidative phosphorylation, aerobic respiration, and cellular respiration. The results of KEGG enrichment analysis of all cluster cells were similar to those of GO enrichment. Oxidative phosphorylation, Huntington disease, chemical carcinogenesis reactive oxygen species, and other related signaling pathways also have strong activity. The expression of transcription factors in individual cell clusters showed that the expression of JUN and FOS associated with the MAPK signaling pathway varied considerably between clusters. Furthermore, we found that the upregulation of FOS would promote the replication of FMDV, whereas the downregulation of FOS would promote the elimination of FMDV. In particular, the host might be able to regulate its own homeostatic relationship with FMDV by modulating the expression of each host factor in its own MAPK signaling pathway to achieve a symbiotic outcome between host and virus. In the recent research, FMDV-persistent infection was found to result in changes in host factor expression in cells, and our results are consistent with the previous research (Han et al., 2018; Han et al., 2021).

In our results, the MAPK signaling pathway was shown to play an important role in the infection of FMDV and in the persistence infection. Indeed, MAPKs, as a group, are capable of being stimulated by different extracellular factors, including cell adhesions, cytokines, hormones, and other. Research has shown that the MAPK signaling pathway is involved in the pathogenesis of human cytomegalovirus (HCMV), and that the ERK and p38 pathways of the MAPK signaling pathway play an important role in the replication cycle of HCMV (Reeves and Compton, 2011), regulating the replication of HCMV by phosphorylating transcription factors that cause transcription of viral and host-associated genes. As feedback, the envelope glycoproteins and other gene expression products of HCMV could also activate the MAPK signaling pathway through different mechanisms, regulating their own and corresponding gene expression of host cells to facilitate HCMV to complete their life cycle and participate in the viral pathogenesis process (Reeves et al., 2012). Similarly, the use of specific inhibitors of the MAPK signaling pathway, either the ERK inhibitor U0126-EtOH, the JNK inhibitor SP600125, or the p38 inhibitor SB203580, was effective in delaying early and late gene transcription of white spot syndrome virus (WSSV) and differentially inhibited WSSV replication (Shi et al., 2012a). WSSV could also regulate the phosphorylation levels of ERK, JNK, and p38, the key kinases in the MAPK signaling pathway (Shi et al., 2012b). Xin et al. found that there were cell heterogeneity, cell inclusions heterogeneity, and cell cycle heterogeneity in the host cells after FMDV infection (Zhang et al., 2013; Xin et al., 2018). We have confirmed by our results from single-cell sequencing that there was also significant cellular heterogeneity in BHK-Op cells, including different clusters of cells. And furthermore, we validated the differential expression of the MAPK signaling pathway and its downstream transcription factors. All these factors could have a dramatic impact on the symbiosis of FMDV virus and host.

**Figure 7:**
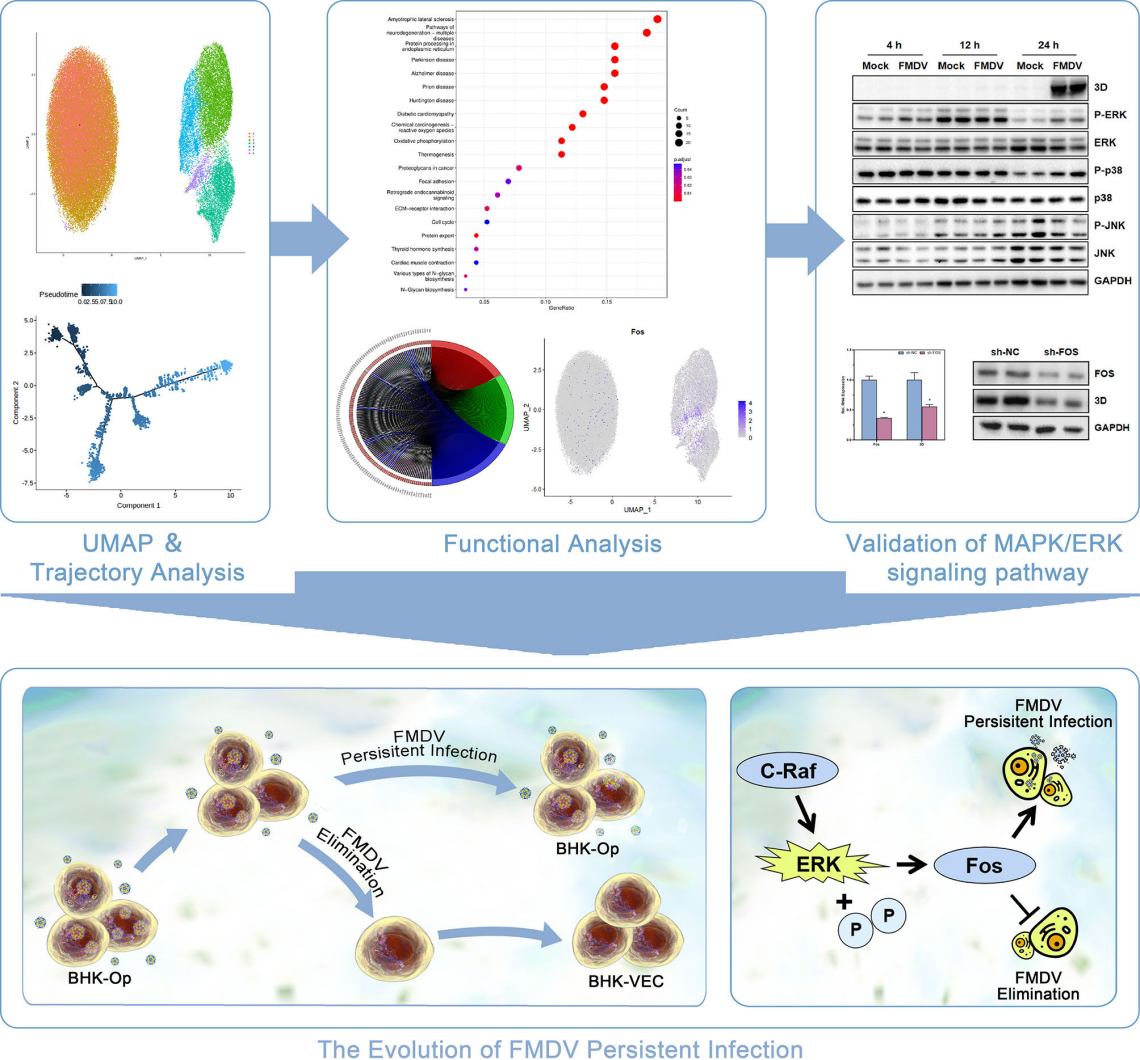
The schematic diagram depicting the regulation of MAPK/ERK signaling pathway on the evolution FMDV-persistent infection.

In conclusion, our research highlights the detailed overview of the evolution of FMDV-persistent infection. Through single-cell sequencing of BHK-Op cells, we found that BHK-Op cells were divided into seven cell clusters, which were two major classes. *Via* pseudotime trajectory analysis, we explained the developmental trajectory of BHK-Op cells that have differentiated from a single-cell cluster to two distinct clusters. GO enrichment analysis, KEGG enrichment analysis, and differential gene analysis were performed on BHK-Op cells, and we found that the MAPK signaling pathway plays an important role in the differentiation of BHK-Op cells. Therefore, we examined the expression of various marker proteins of the MAPK signaling pathway and their phosphorylation levels after FMDV infection. The results revealed that the MAPK/ERK signaling pathway plays an important role in FMDV infection (**Figure 7**). Functional experiments on FOS in BHK-21 and BHK-VEC, which is the downstream of the MAPK/ERK signaling pathway, further confirmed that upregulation of the MAPK/ERK signaling pathway could contribute to virus replication and downregulation could contribute to virus elimination.

## Data Availability Statement

The data presented in the study are deposited in the SRA repository, accession number PRJNA830985.

## Author Contributions

Study design, CS, YY, and XW. Single cell sequencing correlation analyses: YY and XW. Experiments performation, YY, JL, and LH. Data curation, YS, PY, and ZZ. Project administration, CS, YL, and MG. Funding acquisition, CS. Writing - original draft, YY and HD. Writing - review and editing, CS and MG. All authors contributed to the article and approved the submitted version.

## Funding

This work was supported by the financial support of the National Science and Technology Infrastructure Grants (NSTI-CR14-19) and the Science and Technology Innovation Grants of Hubei province (2021CFB357) to CS.

## Conflict of Interest

The authors declare that the research was conducted in the absence of any commercial or financial relationships that could be construed as a potential conflict of interest.

## Publisher’s Note

All claims expressed in this article are solely those of the authors and do not necessarily represent those of their affiliated organizations, or those of the publisher, the editors and the reviewers. Any product that may be evaluated in this article, or claim that may be made by its manufacturer, is not guaranteed or endorsed by the publisher.

## References

[B1] Andreu-Moreno I. Sanjuán R. (2020). Collective Viral Spread Mediated by Virion Aggregates Promotes the Evolution of Defective Interfering Particles. Mbio 11, e02156–e02119. doi: 10.1128/mBio.02156-19 31911487 PMC6946798

[B2] Bankevich A. Nurk S. Antipov D. Gurevich A. A. Dvorkin M. Kulikov A. S.. (2012). SPAdes: A New Genome Assembly Algorithm and its Applications to Single-Cell Sequencing. J. Comput. Biol. 19, 455–477. doi: 10.1089/cmb.2012.0021 22506599 PMC3342519

[B3] Bumgarner R. (2013). Overview of DNA Microarrays: Types, Applications, and Their Future. Curr. Protoc. Mol. Biol. 101, 22.1. doi: 10.1002/0471142727.mb2201s101 PMC4011503 23288464

[B4] Chen S. An G. Wang H. Wu X. Ping P. Hu L.. (2020). Human Obstructive (Postvasectomy) and Nonobstructive Azoospermia–Insights From scRNA-Seq and Transcriptome Analysis. Genes Diseases 9(3), 766–776. doi: 10.1016/j.gendis.2020.09.004 35782978 PMC9243341

[B5] De La Torre J. Martinez-Salas E. Diez J. Villaverde A. Gebauer F. Rocha E.. (1988). Coevolution of Cells and Viruses in a Persistent Infection of Foot-and-Mouth Disease Virus in Cell Culture. J. Virol. 62, 2050–2058. doi: 10.1128/jvi.62.6.2050-2058.1988 2835509 PMC253290

[B6] Domingo E. Escarmiís C. Baranowski E. Ruiz-Jarabo C. M. Carrillo E. Núñez J. I.. (2003). Evolution of Foot-and-Mouth Disease Virus. Virus Res. 91, 47–63. doi: 10.1016/S0168-1702(02)00259-9 12527437

[B7] Fang H. Yuan B. Han L. Xin X. Wang H. Yu F.. (2017). Single-Cell Analysis Reveals the Relevance of Foot-and-Mouth Disease Virus Persistence to Emopamil-Binding Protein Gene Expression in Host Cells. Arch. Virol. 162, 3791–3802. doi: 10.1007/s00705-017-3546-3 28916923

[B8] Gu M. He T. Yuan Y. Duan S. Li X. Shen C. (2021). Single-Cell RNA Sequencing Reveals Multiple Pathways and Tumor Microenvironment Could Lead to Chemotherapy Resistance in Cervical Cancer. Front. Oncol. 4784. doi: 10.3389/fonc.2021.753386 PMC8662819 34900703

[B9] Guo X. Zhang Y. Zheng L. Zheng C. Song J. Zhang Q.. (2018). Global Characterization of T Cells in non-Small-Cell Lung Cancer by Single-Cell Sequencing. Nat. Med. 24, 978–985. doi: 10.1038/s41591-018-0045-3 29942094

[B10] Han L. Xin X. Wang H. Li J. Hao Y. Wang M.. (2018). Cellular Response to Persistent Foot-and-Mouth Disease Virus Infection is Linked to Specific Types of Alterations in the Host Cell Transcriptome. Sci. Rep. 8, 1–13. doi: 10.1038/s41598-018-23478-0 29568077 PMC5864922

[B11] Han L. Yuan Y. Hu J. Li J. Zhu S. Yang P.. (2021). Next-Generation Sequencing Sheds Light on the Interaction Between Virus and Cell During Foot-and-Mouth Disease Virus Persistent Infection. Veterinary Microbiol. 263, 109247. doi: 10.1016/j.vetmic.2021.109247 34649012

[B12] Huang X. Li Y. Fang H. Zheng C. (2011). Establishment of Persistent Infection With Foot-and-Mouth Disease Virus in BHK-21 Cells. Virol. J. 8, 1–12. doi: 10.1186/1743-422X-8-169 21492421 PMC3097150

[B13] Huang X. Li Y. Zheng C.-Y. (2009). A Novel Single-Cell Quantitative Real-Time RT-PCR Method for Quantifying Foot-and-Mouth Disease Viral RNA. J. Virological Methods 155, 150–156. doi: 10.1016/j.jviromet.2008.10.007 19010355

[B14] James A. D. Rushton J. (2002). The Economics of Foot and Mouth Disease. Rev. Scientifique Technique- 21, 637–641. doi: 10.20506/rst.21.3.1356 12523703

[B15] Jerby-Arnon L. Shah P. Cuoco M. S. Rodman C. Su M.-J. Melms J. C.. (2018). A Cancer Cell Program Promotes T Cell Exclusion and Resistance to Checkpoint Blockade. Cell 175, 984–997. e24. doi: 10.1016/j.cell.2018.09.006 30388455 PMC6410377

[B16] Kim D. Kobayashi T. Voisin B. Jo J.-H. Sakamoto K. Jin S.-P.. (2020). Targeted Therapy Guided by Single-Cell Transcriptomic Analysis in Drug-Induced Hypersensitivity Syndrome: A Case Report. Nat. Med. 26, 236–243. doi: 10.1038/s41591-019-0733-7 31959990 PMC7105105

[B17] Kolodziejczyk A. A. Kim J. K. Svensson V. Marioni J. C. Teichmann S. A. (2015). The Technology and Biology of Single-Cell RNA Sequencing. Mol. Cell 58, 610–620. doi: 10.1016/j.molcel.2015.04.005 26000846

[B18] Lavin Y. Kobayashi S. Leader A. Amir E.-A. D. Elefant N. Bigenwald C.. (2017). Innate Immune Landscape in Early Lung Adenocarcinoma by Paired Single-Cell Analyses. Cell 169, 750–765. e17. doi: 10.1016/j.cell.2017.04.014 28475900 PMC5737939

[B19] Li J. Han L. Hao Y. Yuan Y. Wang M. Xin X.. (2020). Comparative Transcriptome Analysis Reveals Different Host Cell Responses to Acute and Persistent Foot-and-Mouth Disease Virus Infection. Virologica Sin. 35, 52–63. doi: 10.1007/s12250-019-00155-8 PMC7035396 31512107

[B20] Maynard A. Mccoach C. E. Rotow J. K. Harris L. Haderk F. Kerr D. L.. (2020). Therapy-Induced Evolution of Human Lung Cancer Revealed by Single-Cell RNA Sequencing. Cell 182, 1232–1251.e22. doi: 10.1016/j.cell.2020.07.017 32822576 PMC7484178

[B21] Pattnaik B. Subramaniam S. Sanyal A. Mohapatra J. K. Dash B. B. Ranjan R.. (2012). Foot-And-Mouth Disease: Global Status and Future Road Map for Control and Prevention in India. Agric. Res. 1, 132–147. doi: 10.1007/s40003-012-0012-z

[B22] Qian S. Fan W. Qian P. Zhang D. Wei Y. Chen H.. (2015). Apigenin Restricts FMDV Infection and Inhibits Viral IRES Driven Translational Activity. Viruses 7, 1613–1626. doi: 10.3390/v7041613 25835532 PMC4411668

[B23] Reeves M. B. Breidenstein A. Compton T. (2012). Human Cytomegalovirus Activation of ERK and Myeloid Cell Leukemia-1 Protein Correlates With Survival of Latently Infected Cells. Proc. Natl. Acad. Sci. 109, 588–593. doi: 10.1073/pnas.1114966108 22203987 PMC3258610

[B24] Reeves M. B. Compton T. (2011). Inhibition of Inflammatory Interleukin-6 Activity *via* Extracellular Signal-Regulated Kinase–Mitogen-Activated Protein Kinase Signaling Antagonizes Human Cytomegalovirus Reactivation From Dendritic Cells. J. Virol. 85, 12750–12758. doi: 10.1128/JVI.05878-11 21937636 PMC3209367

[B25] Salem R. El-Kholy A. A. Ibrahim M. (2019). Eight Novel Single Chain Antibody Fragments Recognising VP2 of Foot-and-Mouth Disease Virus Serotypes A, O, and SAT 2. Virology 533, 145–154. doi: 10.1016/j.virol.2019.05.012 31170612

[B26] Shapiro E. Biezuner T. Linnarsson S. (2013). Single-Cell Sequencing-Based Technologies Will Revolutionize Whole-Organism Science. Nat. Rev. Genet. 14, 618–630. doi: 10.1038/nrg3542 23897237

[B27] Shi H. Yan X. Ruan L. Xu X. (2012a). A Novel JNK From Litopenaeus Vannamei Involved in White Spot Syndrome Virus Infection. Dev. Comp. Immunol. 37, 421–428. doi: 10.1016/j.dci.2012.03.002 22430647

[B28] Shi H. Yan X. Xu X. Ruan L. (2012b). Molecular Cloning and Characterization of a cDNA Encoding Extracellular Signal-Regulated Kinase From Litopenaeus Vannamei. Fish Shellfish Immunol. 33, 813–820. doi: 10.1016/j.fsi.2012.07.008 22884486

[B29] Tang F. Barbacioru C. Wang Y. Nordman E. Lee C. Xu N.. (2009). mRNA-Seq Whole-Transcriptome Analysis of a Single Cell. Nat. Methods 6, 377–382. doi: 10.1038/nmeth.1315 19349980

[B30] Vasudev N. S. Reynolds A. R. (2014). Anti-Angiogenic Therapy for Cancer: Current Progress, Unresolved Questions and Future Directions. Angiogenesis 17, 471–494. doi: 10.1007/s10456-014-9420-y 24482243 PMC4061466

[B31] Wang H. Xin X. Zheng C. Shen C. (2020). Single-Cell Analysis of Foot-And-Mouth Disease Virus. Front. Microbiol. 11, 361. doi: 10.3389/fmicb.2020.00361 32194538 PMC7066083

[B32] Wang C. Yue D. Ma Y. Zhang Q. Li Y. Zhang B.. (2021a). P60. 06 Single Cell Sequencing Analysis Revealed Altered Lung Cancer Microenvironment by Neoadjuvant Immunotherapy. J. Thorac. Oncol. 16, S542–S543. doi: 10.1016/j.jtho.2021.01.965

[B33] Wang W. Zhong Y. Zhuang Z. Xie J. Lu Y. Huang C.. (2021b). Multiregion Single-Cell Sequencing Reveals the Transcriptional Landscape of the Immune Microenvironment of Colorectal Cancer. Clin. Trans. Med. 11, e253. doi: 10.1002/ctm2.253 PMC7775989 33463049

[B34] Xin X. Wang H. Han L. Wang M. Fang H. Hao Y.. (2018). Single-Cell Analysis of the Impact of Host Cell Heterogeneity on Infection With Foot-and-Mouth Disease Virus. J. Virol. 92, e00179–e00118. doi: 10.1128/JVI.00179-18 29444939 PMC5899210

[B35] Zhang H. Li Y. Huang X. Zheng C. (2013). Global Transcriptional Analysis of Model of Persistent FMDV Infection Reveals Critical Role of Host Cells in Persistence. Veterinary Microbiol. 162, 321–329. doi: 10.1016/j.vetmic.2012.09.007 23022682

[B36] Zilionis R. Engblom C. Pfirschke C. Savova V. Zemmour D. Saatcioglu H. D.. (2019). Single-Cell Transcriptomics of Human and Mouse Lung Cancers Reveals Conserved Myeloid Populations Across Individuals and Species. Immunity 50, 1317–1334. e10. doi: 10.1016/j.immuni.2019.03.009 30979687 PMC6620049

