# Supplementary figures and images for: Single-Cell Sequencing Yields Insights in the Evolution of Foot-and-Mouth Disease Virus Persistent Infection
Source: Front Cell Infect Microbiol. 2022 Jul 8;12:940906. doi: 10.3389/fcimb.2022.940906 (PMC9304859; doi:10.3389/fcimb.2022.940906)

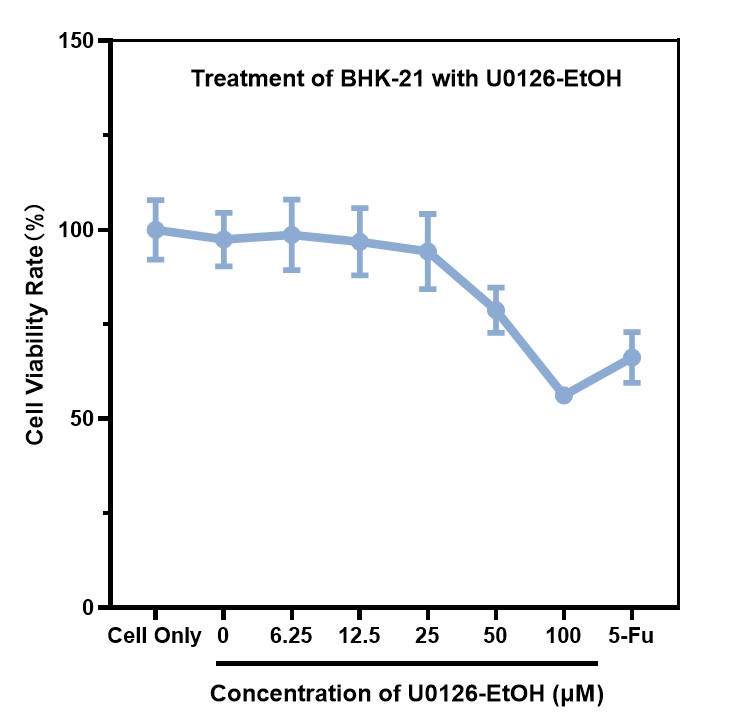

Supplement: Supplementary file 1 [file Image_1.jpeg]

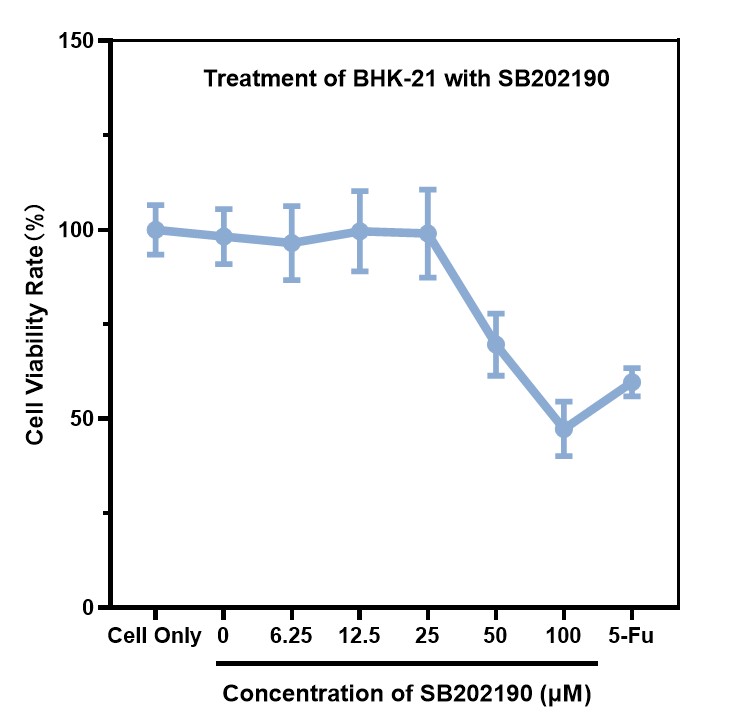

Supplement: Supplementary file 2 [file Image_2.jpeg]
